# Comprehensive molecular characterization of adult H3K27M mutated thalamic glioma long-term survivors

**DOI:** 10.1186/s40164-025-00677-w

**Published:** 2025-06-13

**Authors:** Hao Xu, Xiaomu Hu, Biyun Wang, Ying Sun, Ye Wang, Qisheng Tang, Qiongji Zhu, Kun Song, Hong Chen, Lingchao Chen, Haixia Cheng, Zhiyong Qin

**Affiliations:** 1 Department of Neurosurgery, Huashan Hospital, Fudan University, Shanghai, China; 2 National Center for Neurological Disorders, Shanghai, China; 3 Shanghai Key Laboratory of Brain Function Restoration and Neural Regeneration, Shanghai, China; 4 Department of Pathology, Huashan Hospital, Fudan University, Shanghai, China; 5 Department of Nursing, Huashan Hospital, Fudan University, Shanghai, China; 6 GenomiCare Biotechnology (Shanghai) Co. Ltd, Shanghai, China; 7 Department of Data Science, Shanghai LinZight Technology Co., Ltd, Shanghai, China

**Keywords:** H3K27-altered diffuse midline glioma, Long-term survivor, Mutation analysis, RNA sequencing, DNA methylation array

## Abstract

H3K27-altered diffuse midline gliomas (H3-DMGs) represent aggressive tumors with fatal outcome and exceedingly rare cases have a long-term survival (LTS). We included 5 adult thalamic H3-DMG LTS and 13 short-term survivors (STS), and performed whole exome sequencing, RNA-seq and DNA methylation array. The median overall survival was 48.0 ± 12.1 months for LTS and 12.5 ± 5.9 months for STS. There was no significant difference in clinical characteristics and treatment received between LTS and STS. LTS exhibited more copy number gain and amplification (*P* = 0.007), and tumor microenvironment analysis revealed increased accumulation of M1 macrophage (*P* = 0.005) alongside a notable reduction in cancer-associated fibroblast in LTS (*P* = 0.037). The signatures of LTS and STS were signature 30 (similarity = 76.7%) and signature 6 (81.8%), respectively. Of note, LTS exhibited hypermethylated CpG island (*P* = 0.002). Additionally, we demonstrated that LTS and STS could be distinguished using differentially methylated probes. Collectively, the present study delineated unique molecular characteristics of LTS H3-DMG.

**Supplementary Information:**

The online version contains supplementary material available at 10.1186/s40164-025-00677-w.

**To the editor,**

## Introduction

**Fig. 1:**
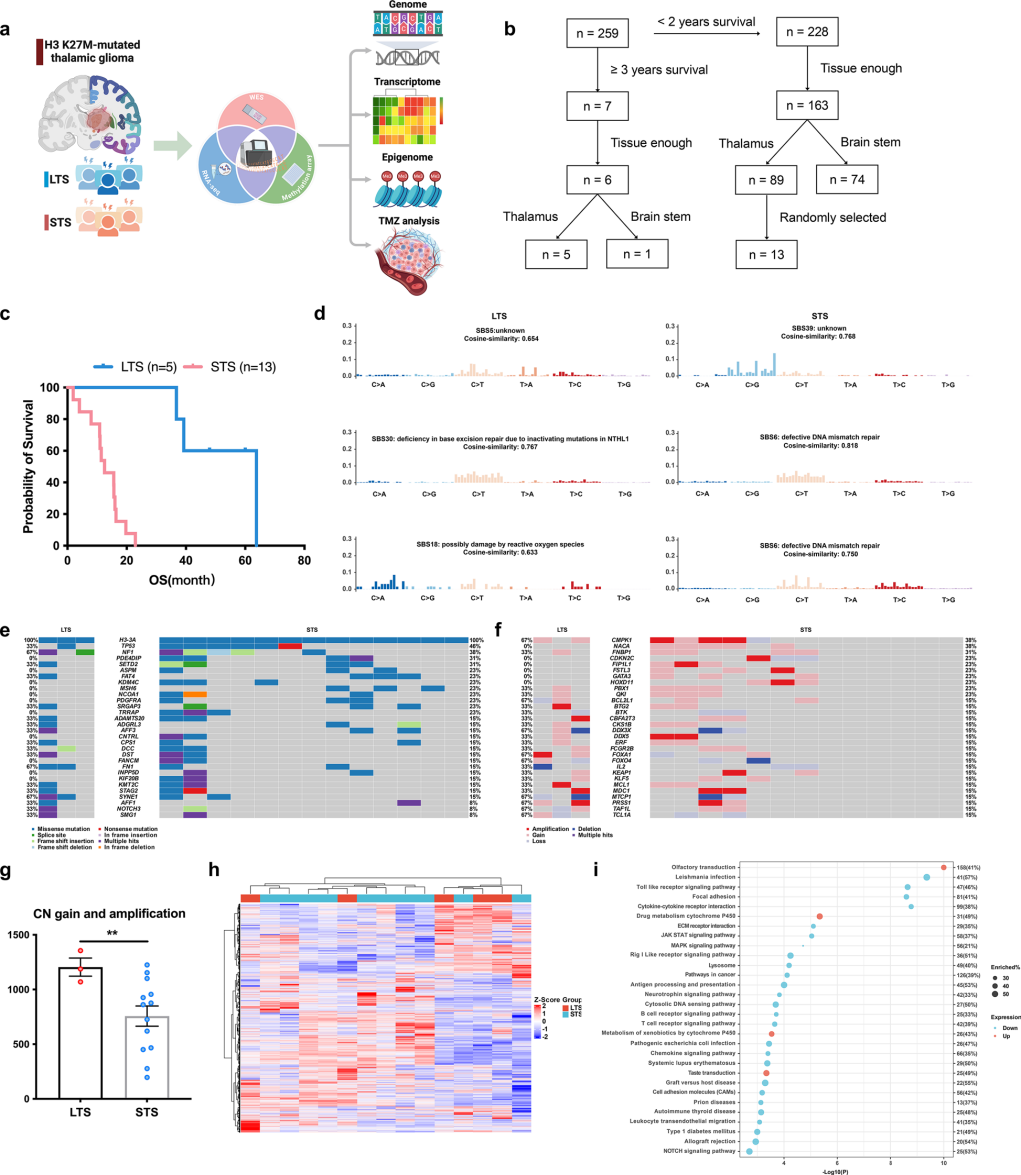
Genomic alteration landscape and transcriptomic difference of LTS and STS. **a** Schematic representation of the integrated multi-omics analytical framework, incorporating genomic, transcriptomic, and DNA methylation modalities to compare LTS and STS cohorts. **b** Flowcharts depicting the patient selection process for the LTS and STS cohorts. Complete inclusion/exclusion criteria are detailed in the “Patient recruitment” section of the Supplementary Materials. **c** Kaplan-Meier survival curves demonstrated a statistically significant OS advantage in the LTS cohort compared to STS counterparts. **d** Cosine similarity analysis revealed distinct mutational patterns, identifying the three most analogous The Single Base Substitution (SBS) signatures for LTS (left panel) and STS (right). **e-f** Oncoplot illustrates the SNV (**e**) and CNV (**f**) patterns between LTS and STS cohorts, with genes ranked by mutation frequency in STS. **g** Dot plot comparing copy number gains and amplifications between LTS and STS cohorts. **h** Heatmap of the top 5,000 most variable genes (standard deviation) displays heterogeneous clustering patterns between LTS and STS cohorts. **i** Pathway enrichment was performed on up-regulated and down-regulated DEGs to identify significantly different biological pathways in LTS versus STS cohorts

H3K27-altered diffuse midline gliomas (DMGs) represent one of the most aggressive intrinsic brain tumors, classified as a distinct entity in the 2016 World Health Organization Classification of Tumors of the Central Nervous System (WHO CNS4) [1]. These tumors predominantly affect pediatric and young adult populations, typically arising in midline structures including the brainstem and thalamus. H3K27-altered DMGs demonstrate poor prognosis with a median overall survival (OS) of just 10–14 months [2, 3]. The exceptionally rare long-term survivors (LTS) of this devastating disease provide a unique opportunity to investigate potential molecular determinants of favorable prognosis. In this study, we present comprehensive molecular profiling of H3K27-altered diffuse thalamic LTS using whole exome sequencing, RNA sequencing, and DNA methylation array (Fig. 1a, Supplementary Fig. S1a).

## Results

Firstly, we identified 259 H3K27M-DMG cases in our center through DNA sequencing. Using a OS threshold of > 36 months, we identified 7 LTS, representing just 2.7% of the total cohort. Among these exceptional LTS cases, 6 had sufficient tissue for multi-omics profiling (5 thalamic and 1 brainstem tumors). The remaining 252 patients included 228 short-term survivors (STS) (OS ≤ 24 months). From 163 STS cases with sufficient tissue (89 thalamic and 74 brainstem tumors), we selected a subset for comparative analysis at random (detailed in Supplementary Material), including 13 thalamic cases (Fig. 1b).

LTS demonstrated substantially prolonged survival (48.0 ± 12.1 months, range 36.8–63.8) versus STS (12.5 ± 5.9 months, range 1.9–22.9) (Fig. 1c, *P* = 0.739). The median age was 32 ± 6.5 years for LTS (range 22–39) and 32 ± 8.3 years for STS (range 22–48) (Table 1, *P* > 0.05). Importantly, there was no obvious difference regarding the treatment received, encompassing surgery and postoperative chemoradiotherapy (Table 1).

**Table 1:** Clinicopathological characteristics of LTS and STS patients

Characteristic	All (*n* = 18)	LTS (*n* = 5)	STS (*n* = 13)	*p*-value
**Gender**				0.490
Female Male	2 16	1 4	1 12	
**Age**,** year**	32 ± 7.7	32 ± 6.5	32 ± 8.3	0.739
**Dominant hemisphere**				0.170
Dominant Nondominant Bilateral	2 15 1	1 3 1	1 12 0	
**Surgery**				0.298
Total resection Subtotal resection Partial resection	4 8 6	1 1 3	3 7 3	
**Treatment**				> 0.999
TMZ + RT TMZ	17 1	5 0	12 1	
**Median OS**,** month**	15.8 ± 18.6	48.0 ± 12.1	12.5 ± 5.9	< 0.001

Signatures of LTS were signatures 30 (similarity = 76.7%, deficiency in base excision repair due to inactivating mutations in *NTHL1*) and 18 (63.3%, possibly damage by reactive oxygen species). By contrast, signature 6 (81.8%, defective DNA mismatch repair) was enriched exclusively in STS (Fig. 1d). *H3-3A* (100%), *NF1* (44%) and *TP53* (44%) demonstrated the highest mutation rate in the entire cohort (Supplementary Fig. S1b). Except for *H3-3A*, *NF1* (67%) and *SYNE1* (67%) demonstrated the highest mutation rate in LTS, while *TP53* (46%) and *NF1* (38%) showed the highest rate of mutation in STS (Fig. 1e).

As for copy number variation (CNV), *CMPK1* (38%), *FNBP1* (31%) and *NACA* (31%) showed the highest rate of CN gain and amplification, while *MTCP1* (25%) and *DDX3X* (19%) demonstrated the highest rate of CN loss and deletion in the entire cohort (Supplementary Fig. S1c). In LTS, *CMPK1* (67%) and *FOXA1* (67%) demonstrated the highest rate of CN gain and amplification, while *MTCP1* (67%) demonstrated the most frequent CN loss and deletion. Considering STS counterparts, *NACA* (38%), *CMPK1* (31%) and *FNBP1* (31%) also demonstrated the top CN gain and amplification, and no CN decrease exceeded 50% (Fig. 1f). However, there was no significant difference in gene mutation and CN change between LTS and STS (Supplementary Fig. S1d-e).

Although LTS and STS were comparable in terms of tumor mutation burden, tumor heterogeneity indicated by mutant-allele tumor heterogeneity (MATH) score, CN loss and general CNV event (Supplementary Fig. S2a), LTS exhibited more CN gain and amplification (Fig. 1g, *P* = 0.007). The top 5000 genes in RNA-seq with highest standard deviation were clustered but failed to distinguish LTS from STS (Fig. 1h). There were 60 down-regulated and 24 up-regulated differentially expressed genes (DEGs) in LTS relative to STS with statistical significance (Supplementary Fig. S2b). Kyoto encyclopedia of genes and genomes (KEGG) pathway enrichment analysis revealed leishmania infection to be the most significantly enriched in down-regulated DEGs, whereas olfactory transduction demonstrated the most significant enrichment in up-regulated DEGs (Fig. 1i, *P* < 0.001).

**Fig. 2:**
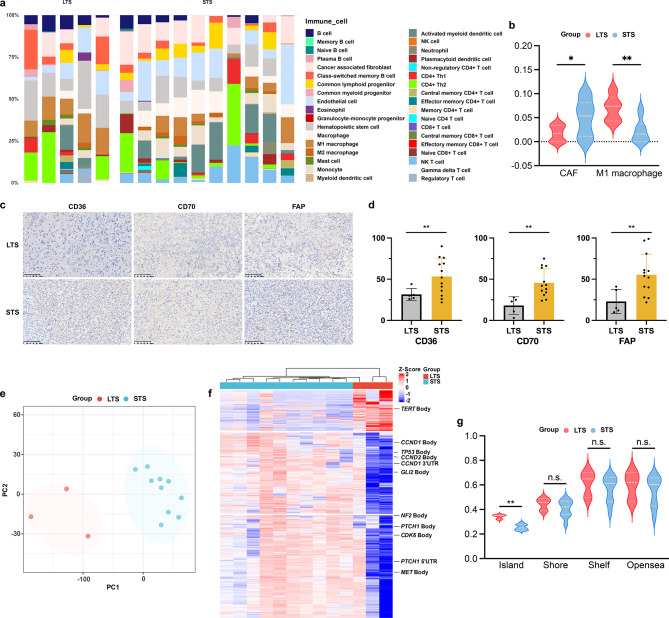
Tumor microenvironment and DNA methylation pattern of LTS and STS. **a** Stacked bar plot displayed immune cell infiltration proportions, with samples grouped by clinical outcome. **b** Violin plot demonstrated significantly lower CAF infiltration and higher M1 macrophage levels in LTS versus STS cohorts. **c** Representative IHC images of CAF markers in LTS (up) and STS (down). **d** Quantification shows significantly reduced CAF markers in LTS. **e** PCA of DMP normalized beta value showed separation between LTS and STS cohorts. **f** Heatmap demonstrated hierarchical clustering of DMPs normalized beta value effectively distinguished LTS from STS cohorts. **e** Violin plot comparing methylation levels (beta value) across CpG genomic contexts (island/shore/shelf/open sea)

We found endothelial cells, hematopoietic stem cells and cancer associated fibroblasts (CAFs) as the predominant infiltrating cells of the entire cohort (Fig. 2a). Notably, LTS exhibited higher infiltration of M1 macrophages (*P* = 0.005) and less infiltration of CAFs in STS (*P* = 0.037) (Fig. 2b). We next performed immunohistochemistry and found the protein levels of CD36 (*P* = 0.006), CD70 (*P* = 0.001) and FAP (*P* = 0.005) were significantly higher in STS (Fig. 2c-d).

Through DNA methylation-based classification, the entire cohort were identified as diffuse midline glioma, H3K27-altered, subtype H3K27-mutant or EZHIP expressing (DMG_K27). A total of 8830 differentially methylated probes (DMPs) were identified comprising of 1598 hyperDMPs and 7232 hypoDMPs (Supplementary Fig. S3a). The detailed DMP distribution is shown in Supplementary Fig. S3b. KEGG analysis of DMPs revealed enrichment of the vibrio cholerae infection (Supplementary Fig. S3c). Principle component analysis (PCA) of DMPs demonstrated a trend of separation (Fig. 2e). Further, hierarchical clustering of DMPs demonstrated clear segregation between LTS and STS cohorts (Fig. 2f). Importantly, the average beta value of LTS was significantly higher than that of STS in CpG island (Fig. 2g, *P* = 0.002).

## Discussion

While H3K27M-DMG LTS showed comparable clinical features to STS, we identified several key molecular distinctions. LTS tumors exhibited a distinct genomic profile characterized by increased CN gain and amplification, mirroring patterns observed in LTS of glioblastoma (GBM) [4]. This CN-enriched signature suggests shared mechanisms of tumor adaptation in rare survivors across different high-grade glioma subtypes.

KEGG of all DEGs demonstrated significant downregulation of the “leishmania infection” pathway in LTS. Mechanistically, this gene set is associated with tumor immune evasion through suppression of antigen presentation and promotion of immunosuppressive macrophage polarization. Notably, recent multi-omics studies have linked leishmania infection-related signatures to poor prognosis in cervical cancer and GBM [5, 6]. The observed suppression of this pathway in LTS patients suggests its potential in conferring survival advantages, possibly via enhanced anti-tumor immunity and reduced metabolic adaptation of malignant cells.

The tumor microenvironment (TME) of LTS exhibited a notable reduction in CAFs alongside increased accumulation of M1 macrophages. Emerging evidence highlights the heterogeneity of CAFs encompassing both tumor-promoting and anti-tumor properties [7]. Specifically, pro-tumorigenic CAF subpopulations marked by FAP, CD36 and CD70 are associated with extracellular matrix remodeling and T-cell exhaustion, driving immunosuppression [7, 8]. Conversely, M1 macrophages, known for their antitumorigenic functions through antigen presentation and pro-inflammatory cytokine production, showed significant enrichment in LTS GBM [4, 9]. Taken together, immune cell infiltration in LTS TME indicated higher antitumor activity compared to that of STS. Still, integrated multi-omics on single-cell level is needed to fully elucidate the complex cellular interactions underlying this distinct immune landscape.

Significantly, our analysis revealed pronounced CpG island hypermethylation in LTS. This finding aligns with emerging evidence demonstrating that global DNA methylation patterns serve as independent prognostic indicators in GBM [10], and corroborates our previous observation of genome-wide hypermethylation in LTS GBM compared to STS [4]. These cumulative findings establish DNA methylation status as a critical molecular determinant with cross-subtype prognostic significance in glioma progression, warranting integration into future molecular classification.

## Conclusion

Despite comparable clinical characteristics to STS, LTS H3-DMG displayed significant molecular divergence in transcriptomics, TME architecture, and epigenetic regulation. Future studies encompassing larger LTS cohorts and spanning multiple centers may advance our understanding of LTS H3-DMG.

## Electronic supplementary material

Below is the link to the electronic supplementary material.

**Supplementary Material 1: Figure 1** SNV and CNV difference between LTS and STS. **(a)** Heatmap displays clinical and multi-omics data for all patients, with lavender indicating missing WES/RNA-seq/methylation data in bottom rows. **(b-c)** Oncoplot showing most frequent somatic SNVs **(b)** and CNVs **(c)** of the entire cohort. Genes are presented in descending order by the mutation rate. d Forest plot of top significant SNVs **(d)** and CNVs **(e)** (ranked by p-value).

**Supplementary Material 2: Figure 2** Genomic and transcriptomic difference between LTS and STS. **(a)** Dot plot provides a quantitative comparison of three key genomic biomarkers, including TMB, MATH score and CNVs between LTS and STS patients. **(b)** Volcano plot displays differentially expressed genes between LTS and STS.

**Supplementary Material 3: Figure 3** DNA methylation pattern of LTS and STS. **(a)** Volcano plot highlighting significant DMPs between LTS and STS cohorts. **(b)** Genomic distribution of DMPs across different CpG gene loci (left) and CpG types (right). **(c)** Dot plot showing KEGG pathways enriched in DMPs.

Supplementary Material 4

## Data Availability

The datasets analyzed for the current study are available from the corresponding author on reasonable request.

## Abbreviations

CAF: Cancer associated fibroblast
CNV: Copy number variation
DEG: Differentially expressed gene
DMG: Diffuse midline gliomas
GBM: Glioblastoma
KEGG: Kyoto encyclopedia of genes and genomes
LTS: Long-term survivors
MATH: Mutant-allele tumor heterogeneity
OS: Overall survival
PCA: Principle component analysis
STS: Short-term survivors
TME: Tumor microenvironment

## References

[1] Louis DN, Perry A, Reifenberger G, von Deimling A, Figarella-Branger D, Cavenee WK, Ohgaki H, Wiestler OD, Kleihues P, Ellison DW. The 2016 world health organization classification of tumors of the central nervous system: a summary. Acta Neuropathol. 2016;131:803–20. 27157931 10.1007/s00401-016-1545-1

[2] Mackay A, Burford A, Carvalho D, Izquierdo E, Fazal-Salom J, Taylor KR, Bjerke L, Clarke M, Vinci M, Nandhabalan M, et al. Integrated molecular Meta-Analysis of 1,000 pediatric High-Grade and diffuse intrinsic Pontine glioma. Cancer Cell. 2017;32:520–e537525. 28966033 10.1016/j.ccell.2017.08.017 PMC5637314

[3] Schreck KC, Ranjan S, Skorupan N, Bettegowda C, Eberhart CG, Ames HM, Holdhoff M. Incidence and clinicopathologic features of H3 K27M mutations in adults with radiographically-determined midline gliomas. J Neurooncol. 2019;143:87–93. 30864101 10.1007/s11060-019-03134-x PMC6482123

[4] Xu H, Chen X, Sun Y, Hu X, Zhang X, Wang Y, Tang Q, Zhu Q, Song K, Chen H, et al. Comprehensive molecular characterization of long-term glioblastoma survivors. Cancer Lett. 2024;593:216938. 38734160 10.1016/j.canlet.2024.216938

[5] Wu Z, Zhuang X, Liang M, Sheng L, Huang L, Li Y, Ke Y. Identification of an inflammatory response-related gene prognostic signature and immune microenvironment for cervical cancer. Front Mol Biosci. 2024;11:1394902. 38903179 10.3389/fmolb.2024.1394902 PMC11187284

[6] Okamoto T, Mizuta R, Demachi-Okamura A, Muraoka D, Sasaki E, Masago K, Yamaguchi R, Teramukai S, Otani Y, Date I, et al. Immune prognostic model for glioblastoma based on the SsGSEA enrichment score. Cancer Genet. 2025;294–295:32–41. 40121844 10.1016/j.cancergen.2025.03.005

[7] Han C, Liu T, Yin R. Biomarkers for cancer-associated fibroblasts. Biomark Res. 2020;8:64. 33292666 10.1186/s40364-020-00245-w PMC7661188

[8] Zhu GQ, Tang Z, Huang R, Qu WF, Fang Y, Yang R, Tao CY, Gao J, Wu XL, Sun HX, et al. CD36(+) cancer-associated fibroblasts provide immunosuppressive microenvironment for hepatocellular carcinoma via secretion of macrophage migration inhibitory factor. Cell Discov. 2023;9:25. 36878933 10.1038/s41421-023-00529-z PMC9988869

[9] Karimi E, Yu MW, Maritan SM, Perus LJM, Rezanejad M, Sorin M, Dankner M, Fallah P, Dore S, Zuo D, et al. Single-cell Spatial immune landscapes of primary and metastatic brain tumours. Nature. 2023;614:555–63. 36725935 10.1038/s41586-022-05680-3 PMC9931580

[10] Eckhardt A, Drexler R, Schoof M, Struve N, Capper D, Jelgersma C, Onken J, Harter PN, Weber KJ, Dive I, et al. Mean global DNA methylation serves as independent prognostic marker in IDH-wildtype glioblastoma. Neuro Oncol. 2024;26:503–13. 37818983 10.1093/neuonc/noad197 PMC10912005

