# Supplementary material for: Comprehensive molecular characterization of adult H3K27M mutated thalamic glioma long-term survivors
Source: Exp Hematol Oncol. 2025 Jun 13;14:84. doi: 10.1186/s40164-025-00677-w (PMC12166572; doi:10.1186/s40164-025-00677-w)
Supplement: Supplementary file 1 — Supplementary Material 1: Figure 1 SNV and CNV difference between LTS and STS. (a) Heatmap displays clinical and multi-omics data for all patients, with lavender indicating missing WES/RNA-seq/methylation data in bottom rows. (b-c) Oncoplot showing most frequent somatic SNVs (b) and CNVs (c) of the entire cohort. Genes are presented in descending order by the mutation rate. d Forest plot of top significant SNVs (d) and CNVs (e) (ranked by p-value). [file 40164_2025_677_MOESM1_ESM.docx]

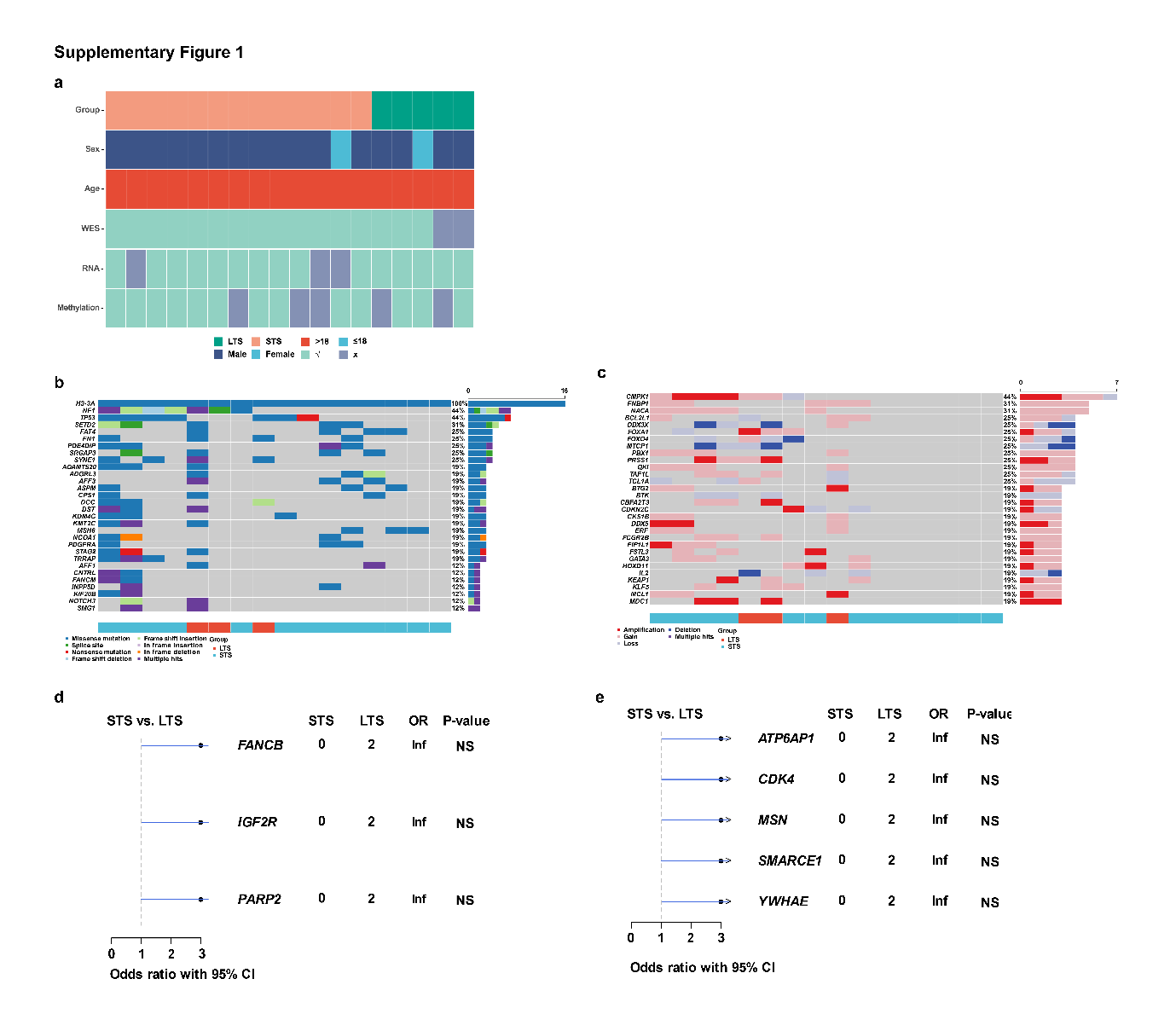


### Supplementary Fig. 1 SNV and CNV difference between LTS and STS

**a** Heatmap displays clinical and multi-omics data for all patients, with lavender indicating missing WES/RNA-seq/methylation data in bottom rows.

**b-c** Oncoplot showing most frequent somatic SNVs (**b**) and CNVs (**c**) of the entire cohort. Genes are presented in descending order by the mutation rate.

**d** Forest plot of top significant SNVs (**d**) and CNVs (**e**) (ranked by p-value).
