# Supplementary material for: Comprehensive molecular characterization of adult H3K27M mutated thalamic glioma long-term survivors
Source: Exp Hematol Oncol. 2025 Jun 13;14:84. doi: 10.1186/s40164-025-00677-w (PMC12166572; doi:10.1186/s40164-025-00677-w)
Supplement: Supplementary file 2 — Supplementary Material 2: Figure 2 Genomic and transcriptomic difference between LTS and STS. (a) Dot plot provides a quantitative comparison of three key genomic biomarkers, including TMB, MATH score and CNVs between LTS and STS patients. (b) Volcano plot displays differentially expressed genes between LTS and STS. [file 40164_2025_677_MOESM2_ESM.docx]

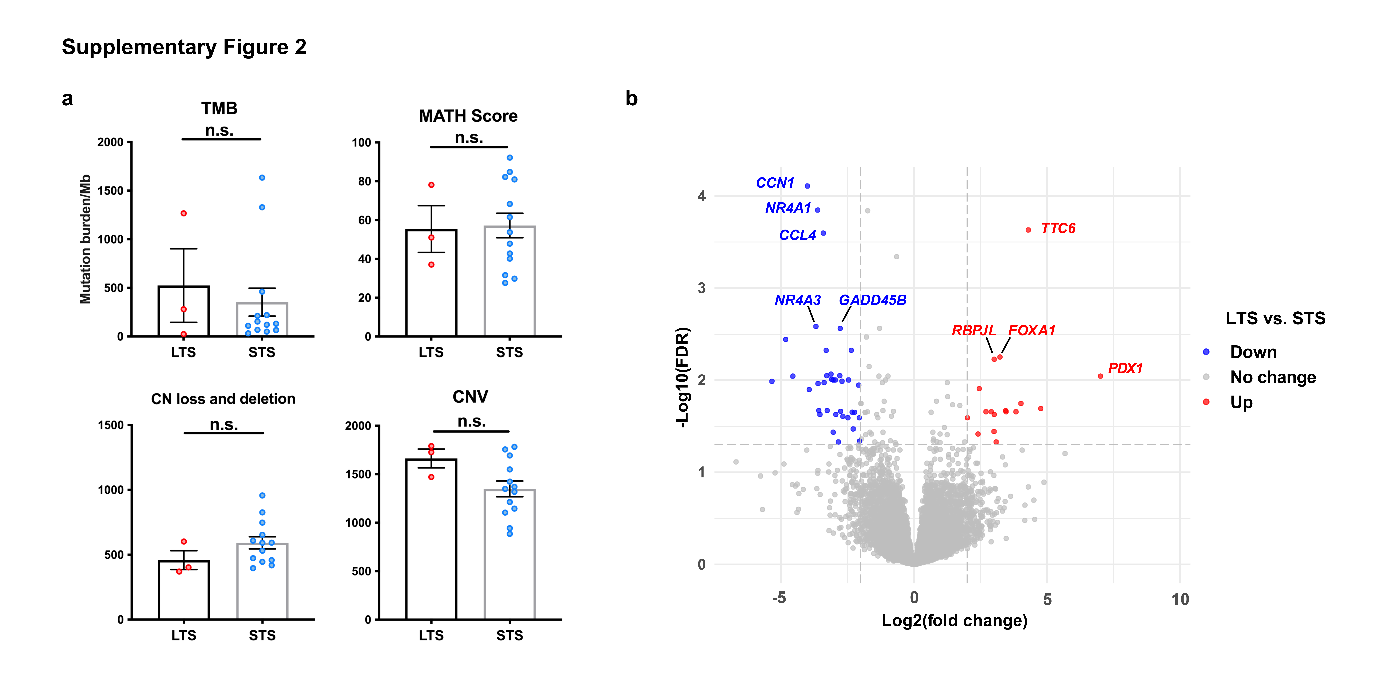


### Supplementary Fig. 2 Genomic and transcriptomic difference between LTS and STS

**a** Dot plot provides a quantitative comparison of three key genomic biomarkers, including TMB, MATH score and CNVs between LTS and STS patients.

**b** Volcano plot displays differentially expressed genes between LTS and STS.
