# Supplementary material for: Comprehensive molecular characterization of adult H3K27M mutated thalamic glioma long-term survivors
Source: Exp Hematol Oncol. 2025 Jun 13;14:84. doi: 10.1186/s40164-025-00677-w (PMC12166572; doi:10.1186/s40164-025-00677-w)
Supplement: Supplementary file 3 — Supplementary Material 3: Figure 3 DNA methylation pattern of LTS and STS. (a) Volcano plot highlighting significant DMPs between LTS and STS cohorts. (b) Genomic distribution of DMPs across different CpG gene loci (left) and CpG types (right). (c) Dot plot showing KEGG pathways enriched in DMPs. [file 40164_2025_677_MOESM3_ESM.docx]

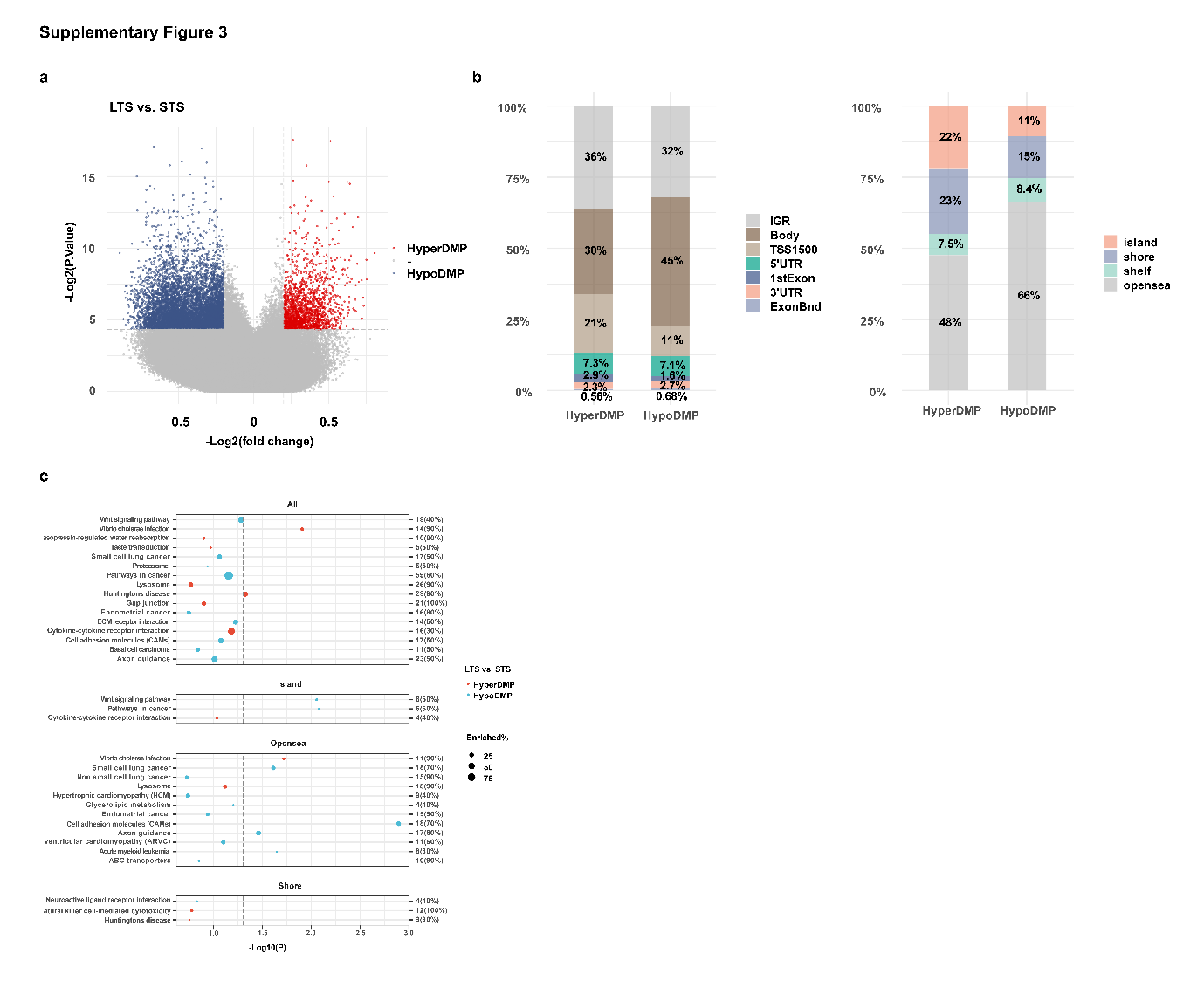


### Supplementary Fig. 3 DNA methylation pattern of LTS and STS

**a** Volcano plot highlighting significant DMPs between LTS and STS cohorts.

**b** Genomic distribution of DMPs across different CpG gene loci (left) and CpG types (right).

**c** Dot plot showing KEGG pathways enriched in DMPs.
