# Supplementary material for: Comprehensive molecular characterization of adult H3K27M mutated thalamic glioma long-term survivors
Source: Exp Hematol Oncol. 2025 Jun 13;14:84. doi: 10.1186/s40164-025-00677-w (PMC12166572; doi:10.1186/s40164-025-00677-w)
Supplement: Supplementary file 4 — Supplementary Material 4 [file 40164_2025_677_MOESM4_ESM.docx]

**Materials and Methods**

**Patient recruitment**

This single-institution study was approved by the Ethics Committee of Huashan Hospital, Fudan University Shanghai, China 200040 (No.KY2015-256). Primary H3-DMG patients who underwent the first surgery at the Department of Neurosurgery, Huashan Hospital between October 2014 and September 2021 were retrospectively analyzed, and recurrent cases were excluded from the present study. Informed consent was signed by each patient preoperatively and all patients agreed to donate their remnant tumor tissue, blood sample and the associated clinical information to Huashan Hospital Standardized Glioma Tissue Bank (GTB) on the premise that the diagnostic procedure and clinical treatment were not compromised by the collection process [1].

OS was defined from the date of surgery to the date of death due to any cause. Patients meeting the following criteria were eligible for the LTS group: (1) OS exceeding 3 years; (2) age 18 or older; (3) histologically diagnosed as DMGs and confirmed as H3K27-altered; (4) tumor available for analysis; (5) tumor located at thalamus；(6) without postoperative Onc201 administration.

Of all 259 patients, 7 exceeded the 3-year OS. Among these patients, 6 possessed sufficient tissue for multi-omics analysis evaluated by an experienced neuropathologist, including 5 thalamus and 1 brain stem tumors (**Fig. 1b**).

Patients in the STS cohort underwent surgery at Huashan Hospital between October 2014 and September 2021 and should meet all the above criteria except for criterion 1 and instead required a less than 24-month OS. Central pathology review was performed by the Department of Pathology based on 2021 WHO CNS5 [2].

228 STS patients were initially identified. Of all 163 STS patients who possessed sufficient tissue, 89 located at thalamus and 13 cases were randomly selected as STS for analysis (**Fig. 1b**).

### Whole exome sequencing (WES)

Cryopreserved blood samples were processed with the Maxwell RSC Blood DNA Kit (AS1400; Promega, WI) on an automated Maxwell RSC system (AS4500, Promega), while formalin-fixed, paraffin-embedded (FFPE) tissues underwent DNA extraction via the NewMag FFPE Tissue DNA Kit (AT3011; Kaitai-Bio, China) with magnetic bead-based purification on a KingFisher Flex platform (Thermo Fisher Scientific, MA). The purified DNA was fragmented to 150-200 bp inserts using a Covaris L220 focused-ultrasonicator (Covaris, MA), followed by exome capture with the VAHTS Core Exome Panel (59.1 Mb; NC001, Vazyme, China). Libraries were constructed using the Universal Plus DNA Library Prep Kit (ND617, Vazyme) featuring dual-index adaptors and PCR amplification, and sequenced on an Illumina NovaSeq 6000 system (CA) configured for 2×150 bp paired-end reads, with real-time base calling managed by RTA3 v3.4.4 software (Illumina). Two LTS samples failed to meet quality control (QC) standards of WES and DNA methylation array due to prolonged storage time (**Supplementary Fig. S1a**).

### Transcriptome Sequencing

FFPE-derived RNA was extracted using the MagMAX FFPE DNA/RNA Ultra Kit (A31881; Thermo Fisher Scientific, MA) on a KingFisher Flex system (ThermoFisher), followed by DNase I treatment. cDNA synthesis was performed sequentially using the NEBNext RNA First Strand Synthesis Module (E7525S; New England Biolabs, MA) and Second Strand Synthesis Modules (E6111S; NEB). Libraries were prepared and sequenced using the same WES protocol (Illumina NovaSeq 6000, 2×150 bp PE).

**Bisulfite sequencing (BS-seq) and methylation-based classification**

Illumina Infinium Methylation 935k BeadChip (Illumina, San Diego, CA, USA) was used for bisulfite sequencing ChIP assay. DNA was isolated from FFPE tissue using DNeasy Blood and Tissue Kit (Qiagen, Hilden, Germany) and genomic DNA was treated with sodium bisulfite using the EZ DNA Methylation kit (ZymoResearch, Irvine, CA, USA) according to manufacturer protocols.

Bioinformatic processing was conducted using the R package ChAMP, with differentially methylated probes (DMPs) defined as those demonstrating absolute deltaBeta > 0.1 at FDR < 0.05 [3]. DMPs were categorized as hyperDMPs (higher average beta-value in LTS compared to STS) or hypoDMPs (vice versa), with methylated CpG sites further annotated by genomic context: island shore (1 to 2,000 bp from island), island shelve (2,001 to 4,000 bp from island) and open sea (> 4,000 bp from island) [4].

Unsupervised hierarchical clustering was performed on the beta values of significant DMPs using Ward’s minimum variance method (ward.D2 algorithm) with a Euclidean distance metric. Samples (columns) were clustered based on their methylation profiles, while probes (rows) were divided into three distinct clusters. Genomic annotations of the probes were displayed according to their chromosomal coordinates and mapped to the nearest gene features.

DNA methylation-based classification was conducted using Heidelberg epignostix CNS tumor classifier v12.8 (https://app.epignostix.com/).

### Data quality control

Following adapter trimming and quality filtering (removing bases with quality scores <20), all sequencing reads were aligned to the GRCh37/hg19 human reference genome using the Burrows-Wheeler Aligner (BWA) algorithm [5]. The quality of data was checked by monitoring the coverage and depths of sequence. RNA-seq was performed with an average depth over 150×, while WES reached an average depth over 190× for tumor samples and over 110 × for normal samples

### Somatic variant identification

Filtered reads were aligned to the hg19 reference genome using BWA-MEM, with germline variants (single nucleotide variation (SNV) and short insertion/deletion (INDEL)) called from blood samples via GATK HaplotypeCaller. Tumor-normal paired somatic variants were detected using GATK Mutect2, followed by functional annotation with VEP (Variant Effect Predictor) [6] and ANNOVAR [7]. We applied stringent quality filters (variant allele frequency (VAF) ≥5%, ≥3 supporting reads) and performed CNV detection using GATK4 CNV tools with custom scripts. Homologous recombination deficiency (HRD) scores were calculated using scarHRD [8], and all final variants were converted to Mutation Annotation Format (MAF) format. Additional analyses included mutant-allele tumor heterogeneity (MATH) score computation (math.score package) [9] and mutation pattern visualization (maftools R package) [10] to assess tumor heterogeneity and gene mutation co-occurrence/exclusivity.

### Tumor mutation burden (TMB)

TMB was calculated as the total count of somatic nonsynonymous mutations (SNV or INDEL) per megabase (mut/Mb) of the sequenced exonic region. This metric was derived by dividing the observed mutation count by the total target size of the SureSelect Human All Exon V7 capture kit (Agilent Technologies), which covers approximately 35 Mb of exonic sequence.

### Copy number variation (CNV)

The normalized depth-of-coverage ratio approach was performed to identify CNV based on WES analysis of paired samples using the GATK somatic-cnvs workflow (version 4.1.4, https://github.com/gatk-workflows/gatk4-somatic-cnvs/tree/master). Copy number was normalized using Z-scores to correct for technical biases (exon size, batch effect, quantity and quality of the sequencing data, local GC content, and genomic mappability). Genes with haploid CN ≤ 1, 1 < CN ≤ 1.2, 3 ≤ CN < 4 and CN ≥ 4 were defined as deletion, loss, gain and amplification, with ≥20% tumor purity required for reliable calling.

### Mutational signature analysis

The Single Base Substitution (SBS) Signatures database (<https://cancer>.sanger.ac.uk/signatures/sbs) was used to calculate the cosine similarity between tumor mutational profile and 99 known SBS signatures. Data analysis was performed using Python-based computational tools: clustering analysis with seaborn package (https://joss.theoj.org/papers/10.21105/joss.03021), t-SNE dimensionality reduction using sklearn package (https://scikit-learn.org/stable/index.html), and visualization with matplotlib (<https://matplotlib.org/stable/index.html>).

### RNA-seq data analysis

RNA-seq reads were aligned to the GRCh37/hg19 reference genome using STAR (v2.2.0.1) [11] and assembled with StringTie2 (v1.3.5) [12] to generate normalized expression matrices in both FPKM (fragments per kilobase of exon per million mapped fragments) and TPM (transcripts per kilobase of exon per million mapped reads) formats. All count data were normalized using the log2-counts per million (log2-CPM) method prior to downstream analysis. Differential expression analysis (DESeq2) [13] identified differentially expressed genes (DEGs) (LTS vs STS; FDR value <0.05, absolute value of log2 fold change > 2), followed by functional enrichment using Kyoto Encyclopedia of Genes and Genomes (KEGG) database [14].

### Immune cell infiltration analysis

TPM-normalized RNA-seq data were analyzed using the R package XCELL to quantify immune cell infiltration scores [15]. The resulting immunophenotypic profiles were subsequently clustered and visualized using ComplexHeatmap [16] and ggplot2 packages [17].

### Gene fusion analysis

### Transcript assembly was performed using StringTie2 (v1.3.5) [12], followed by fusion gene detection with STAR-Fusion (v1.8.0) employing stringent criteria (≥3 supporting reads) [18]. To ensure high-confidence results, putative fusions annotated as "probably false positive" in the FusionHub database were systematically excluded.

### Clustering and principal component analysis (PCA) of expression data

### Consensus clustering was conducted using the R package ConsensusClusterPlus with the following parameters: maximum clusters (maxK) = 7, iterations (reps) = 500, item resampling rate (pItem) = 0.6, feature resampling rate (pFeature) = 1, partitioning around medoids algorithm (clusterAlg = "pam"), and random seed = 10 [19]. PCA was performed using the prcomp function in R to project samples into a two-dimensional space, and the first two PCs were used for plotting.

### *MGMT* promoter methylation

The mean methylation percentages across the 1st to 12th CpG islands were computed, with values exceeding 10% classified as positive.

### Immunohistochemistry (IHC)

All patient specimens were immunostained following the manufacturers’ protocol with the following primary antibodies: CD36 (1:50, 66395-1-Ig, Proteintech), CD70 (1:50, 67749-1-Ig, Proteintech) and FAP (1:50, 27596-1-AP, Proteintech). Whole-slide imaging was conducted using a Vectra automated multispectral microscopy system (Olympus BX53 platform), followed by quantitative image analysis with inForm advanced tissue segmentation software (PerkinElmer).

### Statistics and reproducibility

Data visualization was implemented using the R packages ggplot2, ComplexHeatmap and maftools, along with the Python packages seaborn and matplotlib. Statistical analyses were conducted as follows: categorical variables were evaluated using Pearson's chi-square test, while continuous variables were analyzed with the two-sided Mann-Whitney U test. Survival outcomes were assessed through Kaplan-Meier estimation with between-group comparisons performed using the og-rank test. For pathway analysis, GSEA was executed using the Broad Institute's GSEA software (version 4.3.2, <https://www.gsea-msigdb.org/gsea/doc/GSEAUserGuideFrame.html>).

**References**

1. Aibaidula A, Lu JF, Wu JS, Zou HJ, Chen H, Wang YQ, Qin ZY, Yao Y, Gong Y, Che XM, et al: **Establishment and maintenance of a standardized glioma tissue bank: Huashan experience.** *Cell Tissue Bank* 2015, **16:**271-281.

2. Louis DN, Perry A, Wesseling P, Brat DJ, Cree IA, Figarella-Branger D, Hawkins C, Ng HK, Pfister SM, Reifenberger G, et al: **The 2021 WHO Classification of Tumors of the Central Nervous System: a summary.** *Neuro Oncol* 2021, **23:**1231-1251.

3. Morris TJ, Butcher LM, Feber A, Teschendorff AE, Chakravarthy AR, Wojdacz TK, Beck S: **ChAMP: 450k Chip Analysis Methylation Pipeline.** *Bioinformatics* 2014, **30:**428-430.

4. Timp W, Bravo HC, McDonald OG, Goggins M, Umbricht C, Zeiger M, Feinberg AP, Irizarry RA: **Large hypomethylated blocks as a universal defining epigenetic alteration in human solid tumors.** *Genome Med* 2014, **6:**61.

5. Li H, Durbin R: **Fast and accurate short read alignment with Burrows-Wheeler transform.** *Bioinformatics* 2009, **25:**1754-1760.

6. McLaren W, Gil L, Hunt SE, Riat HS, Ritchie GR, Thormann A, Flicek P, Cunningham F: **The Ensembl Variant Effect Predictor.** *Genome Biol* 2016, **17:**122.

7. Wang K, Li M, Hakonarson H: **ANNOVAR: functional annotation of genetic variants from high-throughput sequencing data.** *Nucleic Acids Res* 2010, **38:**e164.

8. Sztupinszki Z, Diossy M, Krzystanek M, Reiniger L, Csabai I, Favero F, Birkbak NJ, Eklund AC, Syed A, Szallasi Z: **Migrating the SNP array-based homologous recombination deficiency measures to next generation sequencing data of breast cancer.** *NPJ Breast Cancer* 2018, **4:**16.

9. Mroz EA, Tward AD, Hammon RJ, Ren Y, Rocco JW: **Intra-tumor genetic heterogeneity and mortality in head and neck cancer: analysis of data from the Cancer Genome Atlas.** *PLoS Med* 2015, **12:**e1001786.

10. Mayakonda A, Lin DC, Assenov Y, Plass C, Koeffler HP: **Maftools: efficient and comprehensive analysis of somatic variants in cancer.** *Genome Res* 2018, **28:**1747-1756.

11. Dobin A, Davis CA, Schlesinger F, Drenkow J, Zaleski C, Jha S, Batut P, Chaisson M, Gingeras TR: **STAR: ultrafast universal RNA-seq aligner.** *Bioinformatics* 2013, **29:**15-21.

12. Kovaka S, Zimin AV, Pertea GM, Razaghi R, Salzberg SL, Pertea M: **Transcriptome assembly from long-read RNA-seq alignments with StringTie2.** *Genome Biol* 2019, **20:**278.

13. Love MI, Huber W, Anders S: **Moderated estimation of fold change and dispersion for RNA-seq data with DESeq2.** *Genome Biol* 2014, **15:**550.

14. Kanehisa M, Goto S: **KEGG: kyoto encyclopedia of genes and genomes.** *Nucleic Acids Res* 2000, **28:**27-30.

15. Aran D, Hu Z, Butte AJ: **xCell: digitally portraying the tissue cellular heterogeneity landscape.** *Genome Biol* 2017, **18:**220.

16. Gu Z, Eils R, Schlesner M: **Complex heatmaps reveal patterns and correlations in multidimensional genomic data.** *Bioinformatics* 2016, **32:**2847-2849.

17. Wickham H: **ggplot2: elegant graphics for data analysis New York.** *NY: Springer* 2009.

18. Haas BJ, Dobin A, Li B, Stransky N, Pochet N, Regev A: **Accuracy assessment of fusion transcript detection via read-mapping and de novo fusion transcript assembly-based methods.** *Genome Biol* 2019, **20:**213.

19. Wilkerson MD, Hayes DN: **ConsensusClusterPlus: a class discovery tool with confidence assessments and item tracking.** *Bioinformatics* 2010, **26:**1572-1573.
